# Medial and dorsal lateral septum involving social disruption stress-primed escalation in acid-induced writhes

**DOI:** 10.3389/fnmol.2023.1158525

**Published:** 2023-04-20

**Authors:** Yi-Han Liao, Li-Han Sun, Yi-Chi Su, Wei-Jen Yao, Lung Yu

**Affiliations:** ^1^Department of Physiology, College of Medicine, National Cheng Kung University, Tainan, Taiwan; ^2^Division of Cardiology, Department of Internal Medicine, Ditmanson Medical Foundation Chia-Yi Christian Hospital, Chiayi, Taiwan; ^3^Institute of Basic Medical Sciences, College of Medicine, National Cheng Kung University, Tainan, Taiwan; ^4^Ditmanson Medical Foundation Chia-Yi Christian Hospital, Chiayi, Taiwan; ^5^Institute of Behavioral Medicine, College of Medicine, National Cheng Kung University, Tainan, Taiwan

**Keywords:** stress, medial and dorsal lateral septum, nucleus accumbens, TRPV1 receptor, protein kinase C, abdominal pain

## Abstract

**Introduction:**

Stress may cause prospective escalations in abdominal pain magnitude and accumbal TRPV1 expression, while central neural circuits mediating these stress effects remain unclear.

**Methods:**

Using retrograde tracing methods, we first demonstrated the existence of a medial septal-dorsal lateral septal -accumbal circuit very likely involving social disruption stress-primed escalations in acid-induced writhes and accumbal TRPV1 level. An intersectional viral strategy and virus-carrying hM3Dq and hM4Di DREADDs were, then, employed to selectively modulate GABAergic and cholinergic neuronal activity in medial and dorsal lateral septum.

**Results:**

Exciting medial septal GABAergic neuron was found to prevent social disruption stress-primed escalations in acid-induced writhes and accumbal TRPV1 and PKCε expressions. Likewise, inactivating dorsal lateral septal cholinergic neurons was also effective in abolishing these stress-primed escalations. Inactivating GABAergic neuron in non-stressed animals’ medial septum was found to reproduce the stress-primed effects in causing heightened acid-induced writhes and accumbal TRPV1 and PKCε levels.

**Discussion:**

These results, taken together, prompt us to conclude that social disruption stress may produce plastic changes in a newly-identified medial septal-dorsal lateral septal-accumbal circuit. Moreover, medial septal GABAergic hypoactivity and dorsal lateral septal cholinergic hyperactivity are, at least, two likely causes reflecting such stress-produced escalations in abdominal pain magnitude and pain transduction-related protein over-expression in nucleus accumbens.

## 1. Introduction

Individual’s emotional status may affect abdominal pain sensitivity and magnitude (Hockley et al., 2018). Specifically, many investigators report that stress-primed emotional status may enhance abdominal pain magnitude (Nunes-de-Souza et al., 2000; Bielefeldt et al., 2005; Baptista et al., 2009; Langford et al., 2011; Moloney et al., 2015). Not surprisingly, a great number of patients afflicting with stress-related emotion disorders are comorbid with chronic abdominal pain (Fadgyas-Stanculete et al., 2014; Kalinichev et al., 2017; Niraj, 2018). Brain limbic system seems to play a role in mediating the stress-provoked emotional changes and thus prospective escalation of abdominal pain magnitude (Bielefeldt et al., 2005; Fadgyas-Stanculete et al., 2014; DeBerry et al., 2015; Mrozkova et al., 2016; Rossato et al., 2018). Likewise, stress-produced neural plasticity in limbic motivational system may also account for animals’ sensory threshold decreases and/or heightened pain responses (Lee et al., 2015; Greenwood-Van Meerveld and Johnson, 2017). Nonetheless, it is obvious that limbic neuronal circuits and biochemical substrates underlying this stress-primed abdominal pain plasticity are less understood.

Transient receptor potential vanilloid 1 (TRPV1) receptor activation is noticed in nucleus accumbens, a sensory information-integrating structure of the limbic system, in response to noxious tail stimulation (Marinelli et al., 2005). This finding suggests that accumbal TRPV1 receptor may serve as a biochemical substrate of choice to reflect stress stimuli impinging upon the limbic system. Besides, intracellular protein kinase C (PKC) seems to also play a seminal role in modulating TRPV1 sensitization and thus pain magnitude (Than et al., 2013). Using acid-induced writhes to model abdominal pain, we have recently demonstrated that a psychosocial stress may render prospective increases in both accumbal TRPV1 receptor expression and acetic acid-induced writhing magnitude (Liao et al., 2021). And blocking accumbal TRPV1 receptor is found to reliably prevent such stress-primed escalation in acid-induced writhes in the same study (Liao et al., 2021). The latter results prompt us to hypothesize that accumbal TRPV1 and PKC upregulation may be regarded as candidate biomarkers reflecting stress-primed escalation in acid-induced writhes.

In addition to nucleus accumbens, medial and lateral septal nuclei, another two limbic structures, are known for their roles in orchestrating stress-related motivation behavior (Hinman et al., 2018; Tsanov, 2018). For instance, animals’ stress-primed exploration difficulty is, at least in part, attributed to stress-evoked increases in neuronal activity of lateral septal nuclei (Mirrione et al., 2014). Interestingly, tract-tracing studies indicate a sparse and indirect pathway projecting from medial septal nucleus to nucleus accumbens *via* hippocampal relay (Kim et al., 2019; Trouche et al., 2019), although dense and direct lateral septal-accumbal projections are eminent (Ma et al., 2020). Intriguingly, medial and lateral septum seem to be reciprocally connected using behavioral and oscillation analysis methods (Cazala et al., 1988; Tsanov, 2018). One line of evidence suggests that medial septal neuronal activity seems to associate positively with acid-induced writhing magnitude (Liao et al., 2021). Implications embedded in all these results are two-fold. First, there may be existence of a medial septal-lateral septal-accumbal circuit. Second, this hypothetical circuit may account for the stress-caused limbic plasticity and thus stress-primed escalation in acid-induced writhes. Accordingly, this study was undertaken to identify the existence of such medial septal-lateral septal-accumbal circuit. Moreover, we decided to assess the causative relationship between stress-primed neuronal activity plasticity in this circuit, and stress-primed escalations in acid-induced writhes and accumbal TRPV1 and PKC expression.

## 2. Methods

### 2.1. Animals

This study was performed in accordance with the US National Institutes of Health Guide for the Care and Use of Laboratory Animals updated in 2011. Experimental procedures were approved by local Animal Care Committee at National Cheng Kung University College of Medicine (NCKUCM No. 111264). Since female CD-1 mice displayed low rate of aggression toward female C57BL/6 mice, only male mice were included for establishing the social disruption stress model. Male C57BL/6 (8-week-old) mice and CD-1 (at least 6-month-old) retired breeders were obtained from the NCKUCM Laboratory Animal Center (Tainan, Taiwan, ROC) and BioLASCO (Taipei, Taiwan, ROC), respectively. Two weeks prior to the experiments, C57BL/6 (defeated) mice were group housed as a cohort of 3, while CD-1 (aggressor) mice were housed individually in a plastic cage (29 × 19 × 12 cm) in a temperature- and humidity-controlled colony room on a 12-h light/dark cycle with lights on at 0700. All mice had access to food (Purina Mouse Chow, Richmond, IN, United States) and tap water *ad libitum* throughout the experiments. All experiments were conducted from 08:00–16:00 in a temperature (23 ± 1°C)- and humidity (70%)-controlled Laboratory.

### 2.2. Social disruption-induced stress regimen

Social disruption stress regimen was used to model psychosocial stress as previously described (Liao et al., 2021). Male CD-1 aggressors were selected using the criteria that they initiated an attack toward the test C57BL/6 cohort consisting of three male C57BL/6 mice within 3 min for three different cohorts in a row. For each daily aggression episode, a novel CD-1 aggressor was introduced into the home cage of three-C57BL/6 cohort and each C57BL/6 mouse was attacked at least once by the CD-1 aggressor within 30 min. Any C57BL/6 mice in the cohort failed to receive at least one-time offensive attack from novel CD-1 for 2 days in a row, the whole cohort was discarded for further use. Following the aggression episode, C57BL/6 cohort was physically separated by a Plexiglas divider from the CD-1 for the remaining 23.5 h of the day. Six consecutive, daily aggression/separation episodes were conducted for each C57BL/6 cohort (*N* = 3 each). Same CD-1 aggressors were not repeatedly used for each C57BL/6 cohort throughout the 6-day regimen. Three-C57BL/6 residents experiencing no attacks served as non-stressed controls. These control mice were housed in their home cages with a novel C57BL/6 mouse separated by a Plexiglas divider for 6 consecutive days and a novel C57BL/6 conspecific was introduced for each day (Liao et al., 2021).

### 2.3. Acetic acid-induced writhes

Acetic acid-induced writhing response was used to index the magnitude of an acute abdominal pain (Vander Wende and Margolin, 1956) with minor modifications (Liao et al., 2021). In brief, 5 min after an intraperitoneal acetic acid injection (0.9%, 10 ml/kg, Cat# 100063, Merck KGaA, Darmstadt, Germany), stressed and control C57BL/6 mice were individually subjected to an observation arena (29 × 19 × 12 cm). Mouse writhing responses were observed for 20 min in total in the observation arena. The acetic acid-induced writhing response was defined as a stretch of the trunk with or without extension of the limbs, lasting at least 1 s (Liao et al., 2021). Writhing responses were videotaped and rated by trained observers blind to the groups.

### 2.4. Wheat germ agglutinin conjugated to Alexa fluor 594 retrograde tracing methods

Wheat germ agglutinin (WGA) conjugated with Alexa Fluor 594 (AF594) is frequently used to trace tracts in an anterograde and retrograde manner (Nakashima et al., 2000). With the aid of DAPI staining for assuring colocalization of WGA tracer and soma, we were able to identify the localization of WGA retrograde-targeting neuron’s soma. Moreover, using immunostaining methods for labeling specific chemical markers, such as ChAT, vGluT2 and GAD67, we may further character their neurochemical nature for those WGA-targeting somas. Exploiting the aforementioned techniques and rationale, WGA-AF594 was infused into nucleus accumbens (Acb), including medial and lateral part, in an attempt to assess the existence of a potentially existing projection from dorsal lateral septum (LS) to Acb. Likewise, WGA-AF594 was infused into dorsal LS in an attempt to assess the existence of a potentially existing projection from medial septum (MS) to dorsal LS. In this study, stereotaxic surgery was done using consistent anesthesia procedures. In brief, isoflurane anesthesia was initiated in an acrylic glass box pre-flushed with 3–5% isoflurane and maintained *via* a nose nozzle with 2.0–2.5% isoflurane throughout the surgery. WGA-AF594 (Cat# W11262, Thermo Fisher Scientific, Waltham, MA, United States) dissolved in PBS (1 mg/ml, 1 μl/side) was infused into unilateral nucleus accumbens (meidal Acb, AP: + 1.34 mm, ML: 0.75 mm, DV: − 4.50 mm; lateral Acb, AP: + 1.34 mm, ML: 0.30 mm, DV: − 4.50 mm) and dorsal lateral septum (LS, AP: + 0.6 mm, ML: ± 0.3 mm, DV: − 3.3 mm). Infusions were done using a 30-gauge infusion needle and a Hamilton 10-μl microsyringe at a rate of 1 μl/min. After WGA-AF594 infusion completion, the infusion needle was left for an additional 5 min before withdrawal. Three days after the infusion, mice were deeply anesthetized with chloride hydrate (40 mg/kg, Sigma, St. Louis, MO, United States) followed by sodium pentobarbital (80 mg/kg; SCI Pharmtech, Inc., Taoyuan, Taiwan) and transcardially perfused with 0.9% saline, followed by 10% neutral buffered formalin. Mouse brains were removed and postfixed in 10% neutral buffered formalin and subsequently cryoprotected in 30% sucrose solution for 48 h at 4°C. Coronal sections at 20 μm in thickness were made using a microtome (Thermo Scientific CryoStar NX50, Kalamazoo, MI, United States). Quantification of the WGA-AF594-positive neurons was done in LS (AP: ranging 1.10–0.26 mm) and MS (AP: ranging 1.18–0.62 mm). LS was defined by a region encircled by lateral ventricles and a hypothetical line approximately 1 mm below the corpus callosum, while MS referred to an area of 1.25 × 1.5 mm^2^ below LS. Alternate sections from each brain region were used for the immunofluorescent staining. Brain slices were permeabilized and incubated in a blocking buffer (3% BSA and 5% goat or sheep serum in PBST) for 2 h at room temperature. The slices were stained by mouse anti-vGluT2 (1:200; Cat# ab79157, Abcam, Cambridge, United Kingdom), mouse anti-GAD67 (1:200; Cat# MAB5406, Merck KGaA, Darmstadt, Germany), or rabbit anti-ChAT (1:100; Cat# ab6168, Abcam, Cambridge, United Kingdom) for 48 h at 4°C. Brain slices were, then, immunostained with Alexa Fluor 488-conjugated sheep anti-mouse (1:200; Cat# 515-545-062, Jackson ImmunoResearch, West Grove, PA, United States) or Alexa Fluor 488-conjugated goat anti-rabbit (1:200; Cat# 111-545-003, Jackson ImmunoResearch, West Grove, PA, United States) secondary antibodies for 2 h at room temperature, and cell nuclei were counterstained with DAPI (1:5000; Cat# D9542, Sigma-Aldrich, St. Louis, MO, United States). Brain slices were coverslipped and imaged with an Olympus fluorescent microscope (Model: IX71, Olympus, Tokyo, Japan). Z-stack methods were used for processing the presence of fluorescence-positive cells, if not mentioned otherwise. That is, a composite z-stack image was first generated by composing 3–4 projection-compressed images using CellSens software (DP2-BSW ver.2.2, Olympus, Tokyo, Japan) for each 20-μm slice. On those composite z-stack images, fluorescence-positive spots were, then, counted by a rater blind to the grouping. Acb injection correctness was specified by assuring red-illuminating signals were only detected within Acb and the farthest-reaching signals, at best, overpassed 0.1 mm from the border of Acb (Bregma 0.98 mm, Franklin and Paxinos, 2008). Likewise, dorsal LS injection correctness was specified by assuring red-illuminating signals were only detected within dorsal LS and the intactness of lateral ventricle was absolute (Bregma 0.62 mm, Franklin and Paxinos, 2008). Using these criteria, three mice were excluded for quantitative analysis due to their WGA diffusion issues. And a total of 12 (8 for Acb and 4 remaining for dorsal LS) mice were used for assessing the percentage of various chemical neurons originating from dorsal LS and MS.

### 2.5. Viral-mediated gene transfer

In this study, an intersectional viral strategy was used to selectively target and modulate activity of GABAergic and cholinergic neurons in medial and dorsal lateral septum, respectively. That is, inhibitory and excitatory designer receptors exclusively activated by designer drug (hM4Di and hM3Dq DREADDs) and neuronal marker gene-cre recombinase were co-expressed in GABAergic and cholinergic neurons confined to medial and dorsal lateral septum. In this regard, mDlx is regarded as an enhancer exclusively encoded in almost all classes of mouse brain GABAergic neurons (Dimidschstein et al., 2016). To excite medial septal GABAergic neurons, thus, medial septal infusion (a total volume of 0.5 μl) with a mixture of AAV2/9 encoding mDlx-cre recombinase (AAV2/9-mDlx-Cre, 2.84 × 10^12^ gc/ml, Cat# PT-0306, Biohippo, Gaithersburg, MD, United States) and a cre-inducible AAV9 encoding hM3Dq and mCherry (AAV9-hSyn-DIO-hM3Dq-mCherry, 2.4 × 10^13^ gc/ml, Cat# 44361, Addgene, Watertown, MA, United States) were done at the same time. To inhibit medial septal GABAergic neurons, mice received medial septal infusion (a total volume of 0.5 μl) with a mixture of AAV2/9-mDlx-Cre and AAV5-hSyn-DIO-hM4Di-mCherry (2.7 × 10^13^ gc/ml, Cat# 44362, Addgene, Watertown, MA, United States) also at the same time. Viral mixture delivery was done by a 33-gauge dental needle connecting to a polyethylene tubing and a Hamilton 10-μl microsyringe (Nevada, United States) driven by a microdialysis pump at a rate of 0.1 μl/min. Coordinates for targeting medial septum were AP: + 1.00 mm, ML: ± 0.74 mm, and DV: − 5.10 mm at a 10^o^ angle. To inhibit dorsal lateral septal cholinergic neurons, mice received bilateral dorsal lateral septal (AP: +0.6 mm, ML: ±0.3 mm, and DV: −3.3 mm) infusions (0.3 μl/side) with AAV5-hSyn-DIO-hM4Di-mCherry and bilateral accumbal (AP: +1.34 mm, ML: ± 1.00 mm, and DV: − 4.50 mm) infusions of AAV2/9-ChAT-Cre (0.3 μl/side, 2.97 × 10^12^ gc/ml, Cat# PT-0607, Biohippo, Gaithersburg, MD, United States) at the same time. It was of importance to note that two nalbuphine hydrochloride (4 mg/kg/injection, Uni-Pharma Co., LTD, Taipei, Taiwan) injections were intramuscularly given for analgesic purpose.

### 2.6. AAV intersection infection efficiency

For delineating time-dependent infection efficiency, mice receiving intra-medial septal AAV mixture (total volume: 0.5 μl, AAV2/9-mDlx-Cre and AAV9-hSyn-DIO-hM3Dq-mCherry) infusion were deeply anesthetized and transcardially perfused with 0.9% saline, followed by 10% neutral buffered formalin 3 days, 3 and 4 weeks following the AAV mixture infusions. Their brains were removed and postfixed and coronal sections at 20 μm in thickness were done as aforementioned. The slices were stained by rabbit anti-mCherry (1:500, Cat# ab167453, Abcam, Cambridge, United Kingdom) for 48 h at 4°C. Brain slices were immunostained with Alexa Fluor 594-conjugated goat anti-rabbit (1:500, Cat# 111–585-045, Jackson ImmunoResearch, West Grove, PA, United States) secondary antibodies for 2 h at room temperature and cell nuclei were counterstained with DAPI (1:5000; Cat# D9542, Sigma-Aldrich, St. Louis, MO, United States). To assess whether intra-medial septal viral mixture infusions may induce local cell apoptosis, TUNEL (TdT-mediated dUTP nick-end labelling) assay was done (Sun et al., 2023). Formalin-fixed frozen brain sections containing the MS from AAV2/9 and AAV9 mixture-infused mice were collected 3 days after the viral infusions. Brain slice containing kainic acid-treated dorsal auditory cortex was used to serve as a positive control for our TUNEL assay methods. Cell apoptosis was determined with TUNEL Assay Kit - HRP-DAB (Cat# ab206386, Abcam, Cambridge, United Kingdom) per manufacturer’s instructions (Sun et al., 2023).

To reveal infection efficiency of intra-medial septal viral mixture infusion and plausible dose-related off-target effect of C21 treatment (Goutaudier et al., 2020), mCherry and GAD67 staining assays were done. To delineate functional efficiency of such intra-medial septal infusion, lateral septal Fos expression was also quantified. In brief, mice received an intra-medial septal infusion with the AAV mixture (0.5 μl, AAV2/9-mDlx-Cre and AAV9-hSyn-DIO-hM3Dq-mCherry; Supplementary Figure S2A). Mice receiving intra-medial septal viral mixture infusion were divided into two groups with one receiving an intraperitoneal C21 (2 mg/kg) injection and another equivalent volume of saline injection 3 weeks after the infusion. Approximately 90 min after intraperitoneal C21 or saline injection, two groups of mice were deeply anesthetized and their brains were removed and their mCherry expressions were assayed in MS and Fos expression was quantified in their dorsal LS (Liao et al., 2021). For mCherry assays, brain slices were stained by rabbit anti-mCherry (1:500; Cat# 44362, Abcam, Cambridge, United Kingdom) and mouse anti-GAD67 (1:200; Cat# MAB5406, Merck KGaA, Darmstadt, Germany) for 48 h at 4°C. Brain slices were then immunostained with Alexa Fluor 594-conjugated goat anti-rabbit (1:500; Cat# 111-585-045, Jackson ImmunoResearch, West Grove, PA, United States) and Alexa Fluor 488-conjugated sheep anti-mouse (1:200; Cat# 515-545-062, Jackson ImmunoResearch, West Grove, PA, United States) secondary antibodies for 2 h at room temperature and cell nuclei were counterstained with DAPI (1:5000; Cat# D9542, Sigma-Aldrich, St. Louis, MO, United States). For Fos assays, brain slices were stained by rabbit anti-c-Fos (1:2000; Cat# ab190289, Abcam, Cambridge, United Kingdom) for 24 h at 4°C. Slices were, then, immunostained with Alexa Fluor 488-conjugated goat anti-rabbit (1:500; Cat# 111-545-003, Jackson ImmunoResearch, West Grove, PA, United States) secondary antibodies for 2 h at room temperature.

To assess the infection efficacy of inactivating dorsal lateral septum-accumbal cholinergic projection, mice receiving AAV2/9-ChAT-Cre and AAV5-hSyn-DIO-hM4Di-mCherry infusion in nucleus accumbens and AAV5-hSyn-DIO-hM4Di-mCherry (0.3 μl/side) infusion in dorsal LS were used (Supplementary Figure S3A). Coordinates were AP: + 0.6 mm, ML: ± 0.3 mm, DV: – 3.3 mm for dorsal LS and AP: + 1.34 mm, ML: ± 1.00 mm, DV: – 4.50 mm for nucleus accumbens. Three weeks after viral infusions, mice were deeply anesthetized and their brains were removed. The slices were stained by rabbit anti-ChAT (1:100; Cat# ab6168, Abcam, Cambridge, United Kingdom) for 48 h at 4°C. Brain slices were, then, immunostained with Alexa Fluor 488-conjugated goat anti-rabbit (1:200; Cat# 111–545-003, Jackson ImmunoResearch, West Grove, PA, United States) secondary antibodies for 2 h at room temperature, and cell nuclei were counterstained with DAPI (1:5000; Cat# D9542, Sigma-Aldrich, St. Louis, MO, United States).

### 2.7. Double inverted open reading frame (mCherry) localization assay

Histological check was done after the conclusion of the writhing test for each AAV-treated mouse to ensure their viral infection area. Behavioral data of mice with inappropriate AAV-mCherry location (more than 250 μm beyond the aforementioned borders of MS and dorsal LS) were excluded for further analysis. Less than 8% of the viral-infused mice were excluded from analysis due to inappropriate mCherry location in viral infusion experiments. Nine mice were excluded for behavioral analysis, i.e., 3 for experiment of MS infusion with AAV2/9-mDlx-Cre and AAV9-hSyn-DIO-hM3Dq-mCherry in combination, 2 for experiment of MS infusion with AAV2/9-mDlx-Cre and AAV5-hSyn-DIO-hM4Dq-mCherry in combination, and 4 for experiment of dorsal LS AAV5-hSyn-DIO-hM4Di-mCherry infusions and accumbal AAV2/9-ChAT-Cre infusions.

### 2.8. Compound 21 administration

Six-day social disruption stress regimen started 3 weeks after viral infusions for ensuring mice’ comparable transgene expression. Since clozapine metabolite was concerned by multiple injections with clozapine-N-oxide (Thompson et al., 2018), another DREADD agonist, compound 21 (C21), was used in our experiments. For the recombinase-dependent AAV-DREADD on purpose, viral infusion-treated mice were given an intraperitoneal C21 (2 mg/kg, dissolved in saline, Cat# 6422, Tocris Bioscience, Minneapolis, MN, United States; Jendryka et al., 2019; Roselli et al., 2020) and an equivalent volume of saline injection 40 min prior to each daily social disruption session. Such C21 treatment protocol was designed for preventing 6 consecutive days of social disruption-produced plasticity in neuronal activity in the MS-dorsal LS-accumbal circuit. Two days after the conclusion of the social disruption regimen, mice underwent acetic acid-induced writhing experiments and accumbal TRPV1 and PKC assays.

### 2.9. Acetic acid concentration-dependent curve

To gauge whether intra-medial septal viral mixture infusion and C21 treatment alone may affect acetic acid-induced writhing sensitivity, i.e., abdominal pain threshold, acid concentration-dependent curve for writhing response was obtained in viral mixture (AAV2/9-mDlx-Cre and AAV9-hSyn-DIO-hM3Dq-mCherry v.s. AAV2/9-mDlx-Cre and AAV5-hSyn-DIO-hM4Di-mCherry)-, C21- and saline-treated non-stressed mice. Mice undergoing intra-medial septal viral mixture infusion received writhing sensitivity test 4 weeks after the conclusion of the infusion. The remaining mice undergoing intra-medial septal AAV2/9-mDlx-Cre infusion received 6 consecutive C21 and saline injections, respectively, starting 3 weeks after the conclusion of the viral infusion. Approximately 48 h after the conclusion of the 6th C21 and saline injections, these mice’ writhing sensitivity was monitored. Three concentrations, 0.23, 0.45 and 0.90%, of acetic acid were used to obtain the concentration-writhing curve for four treatment groups.

### 2.10. Locomotor activity

To parcel out motor activity impact on writhing observations, animals’ locomotor activity (i.e., the sum of vertical rearing and ambulatory activity) was monitored in a custom-made transparent Plexiglas chamber (29 × 19 × 12 cm) inside the Optovarimax testing apparatus (Columbus Instrument, Columbus, OH, United States) for a total of 10 min (Lai et al., 2008; Ho et al., 2009; Liao et al., 2018). Summation of vertical rearing and ambulation-caused infrared (IR) break count were measured. Two groups of mice were used with one group receiving intra-medial septal viral mixture (AAV2/9-mDlx-Cre and AAV9-hSyn-DIO-hM3Dq-mCherry) infusion and another PBS infusion. Three weeks after the infusion, these mice were further divided into two groups with one group receiving 6 daily intraperitoneal C21 and another saline injection. Two days following the last C21 and saline injection, their locomotor activity was assessed. Same procedure was performed in mice receiving intra-medial septal viral mixture (AAV2/9-mDlx-Cre and AAV5-hSyn-DIO-hM4Di-mCherry) infusion.

### 2.11. Western immunoblotting for accumbal TRPV1, PKCα and PKCε expression

We decided to assess the long-term impact of stress and chemo-genetically-altered neuronal activity in this medial septal-dorsal lateral septal circuit on accumbal TRPV1 and PKC, a TRPV1 response-enhancing protein kinase, proteins (Gu et al., 2018). Mice were euthanized approximately 3 days after the conclusion of the last C21 and saline injection. Their brains were removed and placed on the dorsal surface on a glass dish sitting on crushed ice. Coronal brain slices (at 1 mm in thickness) were made using a mouse brain slicer matrix (Zivic Instruments, Pittsburgh, PA, United States). A portion of bilateral nucleus accumbens sample was punched out from the respective slabs using a 1-ml pipette tip cutting to an internal diameter of 0.7 mm. The tissue samples were homogenized in ice-cold lysis buffer containing protease inhibitor cocktail (APExBIO Technology, Houston, TX, United States). Homogenates were centrifuged at 13,500 rpm for 10 min at 4°C, and the protein concentrations of supernatants were determined by Bradford method (Bio-Rad Protein Assay, Cat# 5000006, BioRad Laboratories, Hercules, CA, United States) using bovine serum albumin as standards. Samples were heated for 5 min at 95°C in 2 × sample buffer. The electrophoretically separated proteins were transferred to PVDF membranes (Thermo Fisher Scientific, Waltham, MA, United States), blocked with 5% non-fat milk in phosphate saline-Tween-20. The membranes were then incubated overnight at 4°C with the primary antibody, including mouse-anti-mouse TRPV1 (1:200; Cat# ab203103, Abcam, Cambridge, MA, United States), rabbit-anti-mouse PKCα (1:200, Thermo Fisher Scientific, Waltham, MA, United States), rabbit-anti-mouse PKCε (1:500; Cat# ab63638, Abcam, Cambridge, United Kingdom) and mouse anti-β actin antibody (1:10,000; Cat# MAB1501, Merck Millipore; Middlesex County, MA, United States). Following incubation with sheep anti-mouse and goat anti-rabbit secondary antibody (from Jackson ImmunoResearch Laboratories; West Grove, PA, United States), the blots were developed with ECLTM Western blot detection kit (Cat# PK-NEL105001EA, Perkin-Elmer^™^ Life Sciences, Boston, MA, United States). Densitometry was conducted using GeneSys image capture software and ImageJ image analysis software.

### 2.12. Statistical analysis

All analyses were conducted by exploiting Prism 7. To assess the effects of enhancing medial septal GABAergic inhibition on dorsal lateral septum Fos expression, an unpaired *t*-test was used. To assess the effects of enhancing medial septal GABAergic inhibition on stress-primed writhing escalation and accumbal TRPV1 and PKCε overexpression, a two-way (no stress vs. psychosocial stress pre-exposure, and saline vs. C21 administration) ANOVA was used. Likewise, a two-way (viral mixture vs. PBS and C21 vs. saline) ANOVA was used to assess plausible modulating effects of viral mixture and C21 treatment on animals’ locomotor activity. A two-way ANOVA (three acid concentrations and three treatments) was used to assess concentration-dependent curve alterations in viral mixture-, C21- and saline-treated mice. A two-way (stress pre-exposure vs. no stress, and saline vs. C21 administration) ANOVA was used to determine the effect of dampening medial septal GABAergic inhibition on acid-induced writhing magnitude and accumbal TRPV1 and PKCε expression. A two-way (no stress vs. stress pre-exposure, and saline vs. C21 administration) ANOVA was used to examine the effect of inactivating dorsal lateral septal-accumbal cholinergic projections on stress-primed writhing escalation and accumbal TRPV1 and PKCε overexpression. Bonferroni’s *post hoc* comparisons were employed if appropriate. All the level of statistical significance was set at *p* < 0.05.

## 3. Results

### 3.1. Wheat germ agglutinin-AF594 retrograde tracing results revealed a medial septal-dorsal lateral septal-accumbal projection

**Figure 1:**
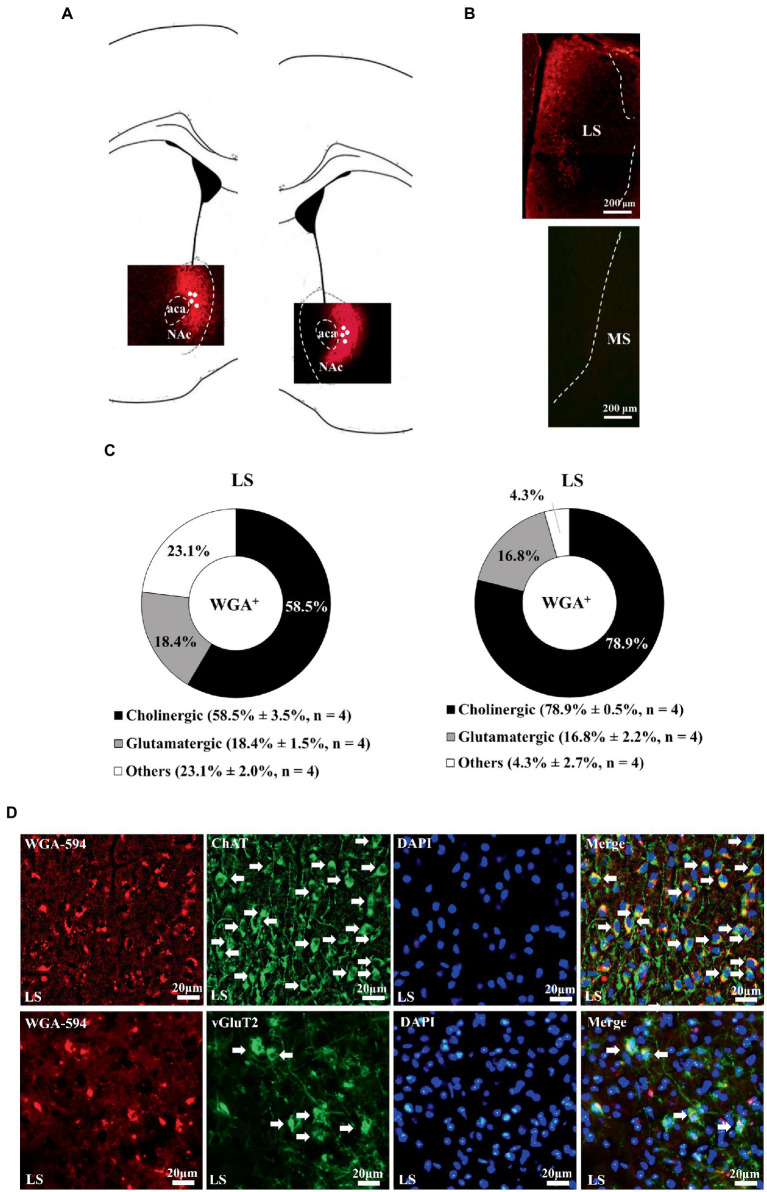
Unilateral accumbal WGA-AF594 infusion was retrograde traced to cholinergic neurons in ipsilateral dorsal lateral septum. **(A)** Schematics of nucleus accumbens (Acb) and representative unilateral WGA-AF594 infusion (red) sites in medial and lateral part of Acb. Solid white circles stand for injection site. **(B)** Representative photomicrographs showing WGA-positive (red) spots in lateral septum (LS) but not medial septum (MS). **(C)** Pie charts for cholinergic, glutamatergic and other neurons of WGA-positive spots in dorsal lateral septum in intra-medial (left panel) and lateral (right panel) Acb WGA infusions. Mean ± SEM is shown. **(D)** Upper panel: representative photomicrographs of WGA + (red), ChAT + (green), DAPI + (blue), and (WGA/ChAT/DAPI) + (indigo center, yellow surround) neurons in lateral septum. Lower panel: representative photomicrographs of WGA + (red), vGluT2+ (green), DAPI + (blue), and (WGA/vGluT2/DAPI) + (indigo center, yellow surround) neurons in lateral septum. White arrows annotate positive cells. ChAT and vGluT2 are short forms of choline acetyl-transferase and vesicular glutamate transporter 2. Scale bar = 20 μm.

**Figure 2:**
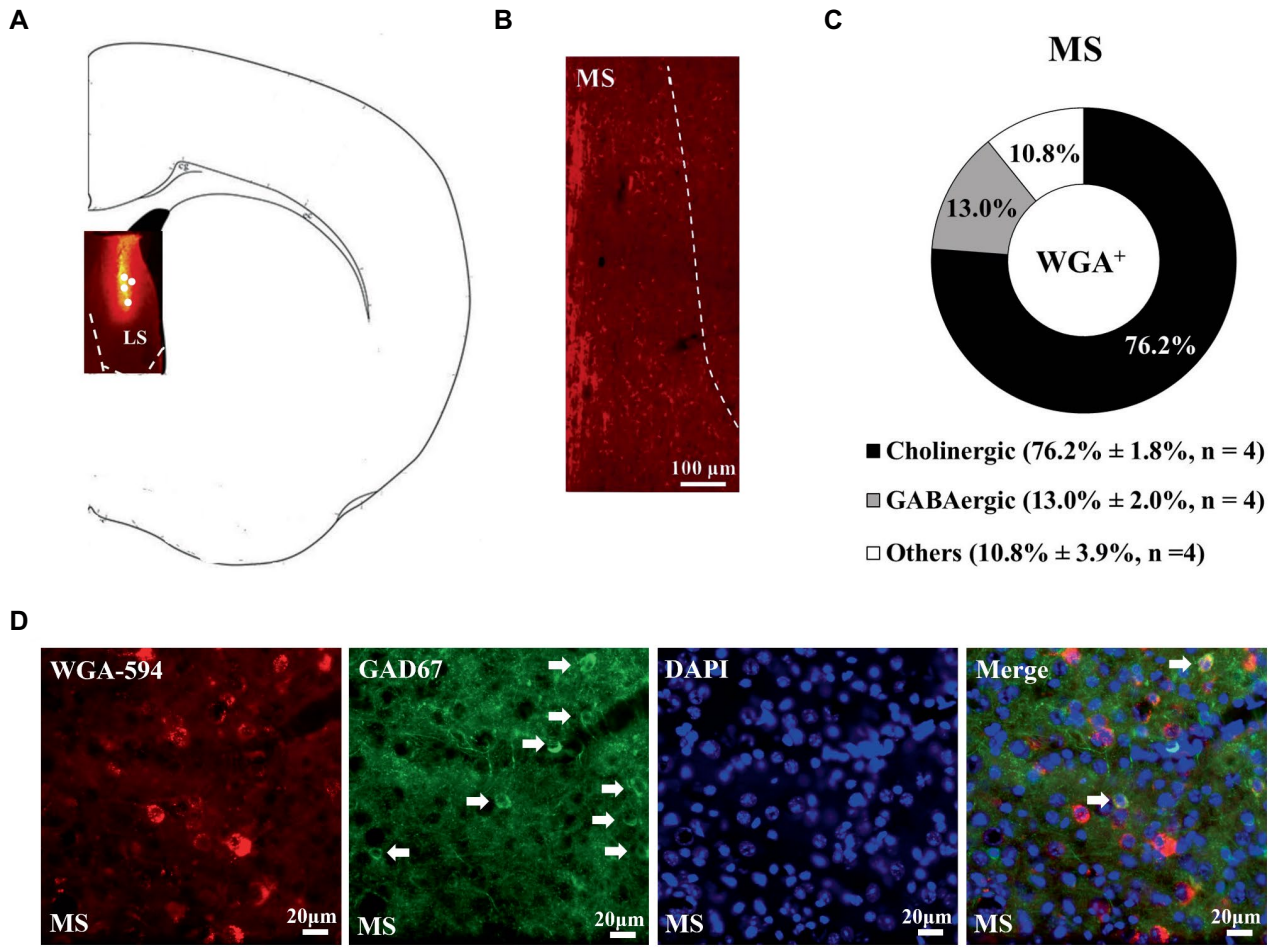
Unilateral dorsal lateral septal WGA-AF594 infusion was retrograde traced to GABAergic neurons in ipsilateral dorsal lateral septum. **(A)** Schematics of lateral septum and representative WGA-AF594 infusion (red) site in dorsal lateral septum. Solid white circles represent injection site. **(B)** Representative photomicrograph showing WGA-positive (red) spots in medial septum (MS). **(C)** Pie diagram for cholinergic, GABAergic and other neurons of WGA-positive spots in medial septum. Mean ± SEM is shown. **(D)** Representative photomicrographs of WGA + (red), GAD67 + (green), DAPI + (blue), and (WGA/GAD67/DAPI) + (indigo center, yellow surround) neurons in medial septum. White arrows annotate positive cells. Scale bar = 20 μm.

Wheat germ agglutinin-AF594 injection confined to the nucleus accumbens, including the medial and lateral parts, produced retrograde neuronal labeling in the lateral, but not medial, septal nucleus, supporting the scenario LS neurons are one of the afferent sources for nucleus accumbens (Figure 1). Moreover, it was of importance to note that all WGA/DAPI co-staining spots were specifically confined in the dorsal part of the LS in this regard. Of all WGA-AF594 and DAPI co-labelled neurons in dorsal LS, 58.5% ± 3.5% (originated from the medial part of nucleus accumbens) and 78.9% ± 0.5% (originated from the lateral part of nucleus accumbens) were choline acetyl-transferase (ChAT)-immunoreactive (that is, cholinergic), 18.4% ± 1.5% (from medial part of nucleus accumbens) and 16.8% ± 2.2% (from lateral part of nucleus accumbens) were vesicular glutamate transporter 2 (vGluT2)-immunoreactive (i.e., glutamatergic), and 23.1% ± 2.0% (from medial nucleus accumbens) and 4.3% ± 2.7% (from lateral nucleus accumbens) were other neurons (4 mice for each; Figure 1). When WGA-AF594 infusions (1 μl/side) was done in the dorsal part of the LS, we identified MS as one of the afferent sources for dorsal LS for the first time (Figure 2). Of all WGA-AF594 and DAPI positive neurons in MS, 76.2% ± 1.8% were cholinergic, 13.0% ± 2.0% glutamate decarboxylase (GAD67)-immunoreactive (i.e., GABAergic), and 10.8% ± 3.9% other neurons (4 mice for each; Figure 2).

### 3.2. Enhancing MS GABAergic activity prevented stress-primed writhing escalation and accumbal TRPV1 and PKCε over-expression

For delineating time-dependent viral infection efficiency, mice undergoing an intra-medial septal viral mixture infusion (AAV2/9-mDlx-Cre and AAV9-hSyn-DIO-hM3Dq-mCherry) were used 3 days, 3 and 4 weeks following such infusion. We first showed that intra-MS infusion (a total volume of 0.5 μl) with AAV9-hSyn- DIO-hM3Dq-mCherry (2.4 × 10^13^ gc/ml) alone failed to show mCherry presence at 3 weeks following the viral infusions (Supplementary Figure S1A). Moreover, intra-MS viral mixture infusion did not yield observed mCherry expressions 3 days after the infusions (Supplementary Figure S1B). Most importantly, intra-MS viral mixture infusion yielded eminent and comparable mCherry expressions in GABAergic neurons at 3 and 4 weeks after the viral mixture infusion (Supplementary Figure S1B). Finally, we demonstrated that our intra-MS viral mixture infusion did not cause observable cell apoptosis in local infusion area (Supplementary Figure S1C).

To further assess viral mixture infection efficiency and plausible C21 dose-related off-target effect (Langford et al., 2011), MS mCherry and GAD67 co-staining assays were done in intra-MS viral mixture-infused mice. To delineate functional efficiency of such intra-MS infusion, dorsal LS Fos expression was quantified. All mice received an intra-MS infusion with AAV mixture (a total volume of 0.5 μl, AAV2/9-mDlx-Cre and AAV9-hSyn-DIO-hM3Dq-mCherry; Supplementary Figure S2A). Three weeks after viral mixture infusion, mice received a single C21 (2 mg/kg) injection. Approximately 63.2 ± 3.7% of MS GABAergic neurons were also mCherry-positive, suggesting an acceptable infection efficiency with this viral mixture method. Moreover, approximate 76.1 ± 3.5% mCherry-positive cells were GABAergic neurons using such methods, suggesting low off-target effect of intra-MS viral mixture infusion and intraperitoneal C21 treatment (a single dose of 2 mg/kg; Supplementary Figures S2B,C). As for functional efficiency assessment, mice receiving intra-MS viral mixture infusion were divided into two groups with one group receiving a C21 (2 mg/kg) injection and another equivalent volume of saline injection 3 weeks after the viral mixture infusion. Forty minutes after C21 injection, mice receiving C21 injection were noticed to have reliable decline in dorsal LS Fos expression as compared to the mice receiving saline injection (Supplementary Figure S2D). The latter results suggest MS GABAergic neurons indeed send their inhibitory control onto neurons in dorsal LS.

**Figure 3:**
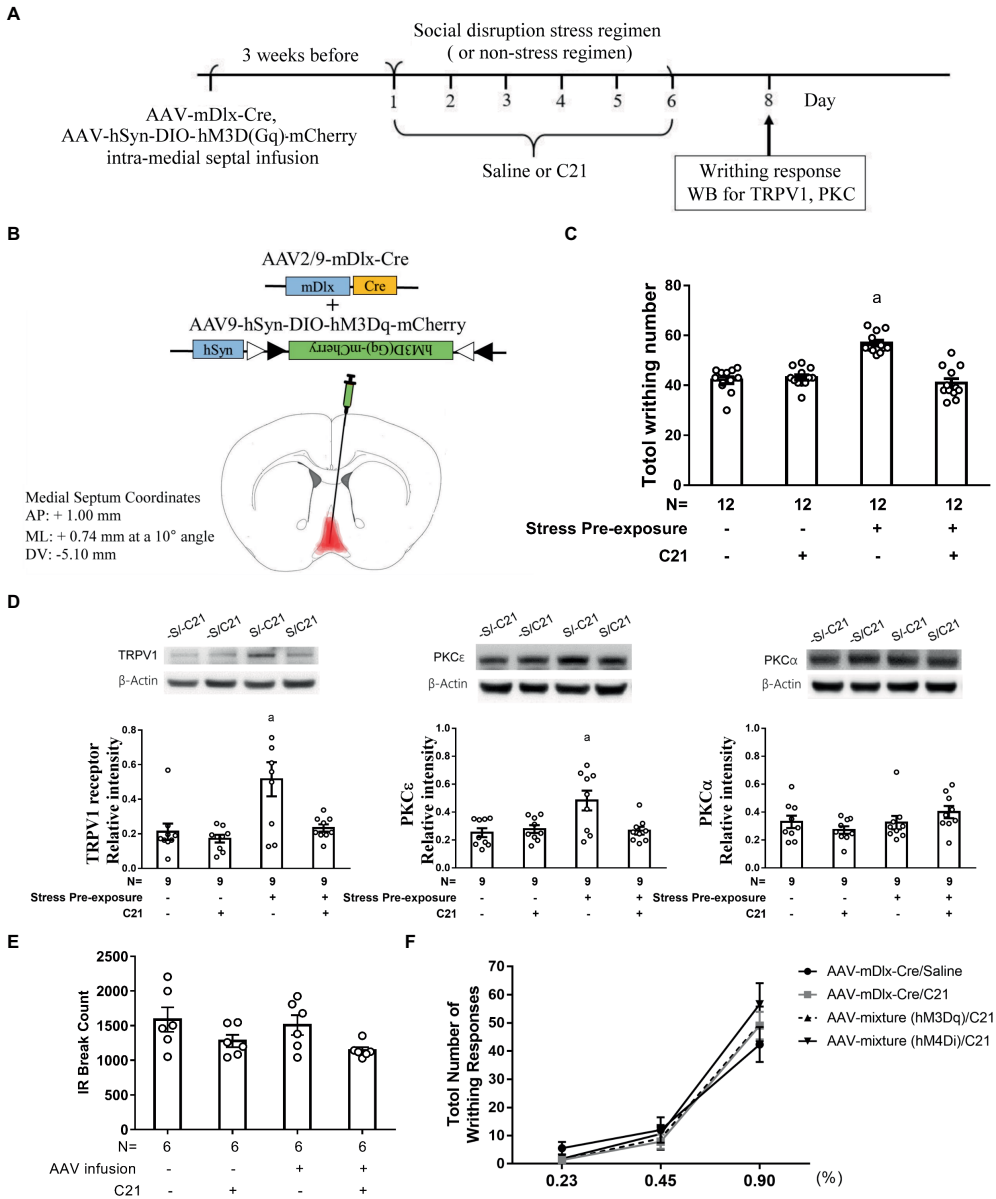
Enhancing medial septal GABAergic activity prevented stress-primed writhing escalation and accumbal TRPV1 and PKCε over-expression. **(A)** Timeline for viral infection, C21 treatment, the stress regimen, writhing response test and Western assay. **(B)** Intra-medial septal viral mixture infusion. Viral mixture with a total volume of 0.5 μl consists of AAV2/9 encoding mDlx-cre recombinase (2.84 × 10^12^ gc/ml) and a cre-inducible AAV9 encoding hM3Dq and mCherry (2.4 × 10^13^ gc/ml). Overlayed red shaded area indicates all confirmed AAV-mCherry labeling results. **(C)** Total acetic acid-induced writhing responses. ^a^Significantly greater than the remaining three groups. **(D)** Accumbal TRPV1, PKCε and PKCα level. ^a^Significantly greater than the remaining three groups. **(E)** No effects of C21 administration or intra-medial septal viral infusion on animals’ locomotor activity. **(F)** Effects of C21 administration or intra-medial septal viral infusion and C21 in combination on acid concentration-dependent curve. [AAV-mixture (hM3Dq): AAV-mDlx-Cre and AAV-hSyn-DIO-hM3Dq-mCherry in combination; AAV-mixture (hM4Di): AAV-mDlx-Cre and AAV-hSyn-DIO-hM4Di-mCherry in combination]. Graphs show mean ± SEM.

While cholinergic neurons outnumbered GABAergic neurons in MS-dorsal LS projection, activating medial septal GABAergic efferent is predominant in its role in dampening stimulant-induced behavioral agitation (Bortz and Grace, 2018). Psychosocial stress exposure is found to associate positively with MS neuronal hyperactivity (Liao et al., 2021). In contrast, inhibiting medial septal neuronal activity may prevent stress-primed escalations in acetic acid-induced writhes and accumbal TRPV1 expression (Liao et al., 2021). Thus, we decided to assess whether the MS-dorsal LS GABAergic projection may play a critical role in priming for this prospective stress-primed behavioral and biochemical plasticity. To this end, stressed and non-stressed control mice were used and their MS GABAergic neurons were exclusively targeted to express membrane hM3Dq receptor (see “Methods” for details). One half of mice in stressed and control groups received C21 treatment (for a total of six injections), while the remaining half from each group received equivalent volume of saline injections (Figures 3A,B). These animals’ acetic acid-provoked writhing responses and accumbal TRPV1 and PKCα and PKCε expression were gauged. To avoid acid treatment and writhing behavior confounds on accumbal TRPV1 and PKC isoform assays, mice being used for writhing test were not repeated used for biochemical assay. A two-way (stress pre-exposure × C21) ANOVA revealed an interactive effect of stress pre-exposure and C21 administration on total writhing number [*F*(1,44) = 38.2, *p* < 0.0001] (Figure 3C). Post-hoc tests further revealed that 6 consecutive days of daily intraperitoneal C21 injection (2 mg/kg each) did not seem to affect acid-provoked writhes (Figure 3C). Six days of stress pre-exposure was found to produce acid-provoked writhing escalation, while such escalation was prevented by C21 treatment (Figure 3C). Moreover, a two-way ANOVA indicated that there was an interactive effect of stress pre-exposure and C21 administration on accumbal TRPV1 expression [*F*(1,32) = 4.519, *p* = 0.0413] (Figure 3D). Social disruption stress pre-exposure produced significant increases in TRPV1 expression in nucleus accumbens (Figure 3D). In contrast, such increase was prevented by daily C21, but not saline, administration (Figure 3D). Furthermore, accumbal PKCε level was elevated by the stress pre-exposure, while such elevation was prevented by C21 treatment [*F*(1,32) = 6.392, *p* = 0.0166] (Figure 3D). Regardless of C21 administration and stress pre-exposure, accumbal PKCα level was unaltered (Figure 3D). It was of importance to note that using these viral mixture methods to enhance MS GABAergic neural activity did not alter animals’ locomotor activity (Figure 3E). To parcel out the possibility that intra-MS viral mixture infusion and C21 may affect mice’ abdominal pain threshold, an acid dose-dependent curve was obtained. We found that our viral mixture infusion and C21 treatment did not seem to affect acetic acid-induced writhing sensitivity and threshold (Figure 3F). These results, taken together, suggest that activating MS GABAergic neurons did not affect animals’ general motor activity or their abdominal pain sensitivity, while prevented stress-primed writhing escalation and accumbal TRPV1 and PKCε over-expression.

### 3.3. Dampening MS GABAergic activity produced stress priming-like effect in acid-induced writhes and accumbal TRPV1 and PKCε expression

**Figure 4:**
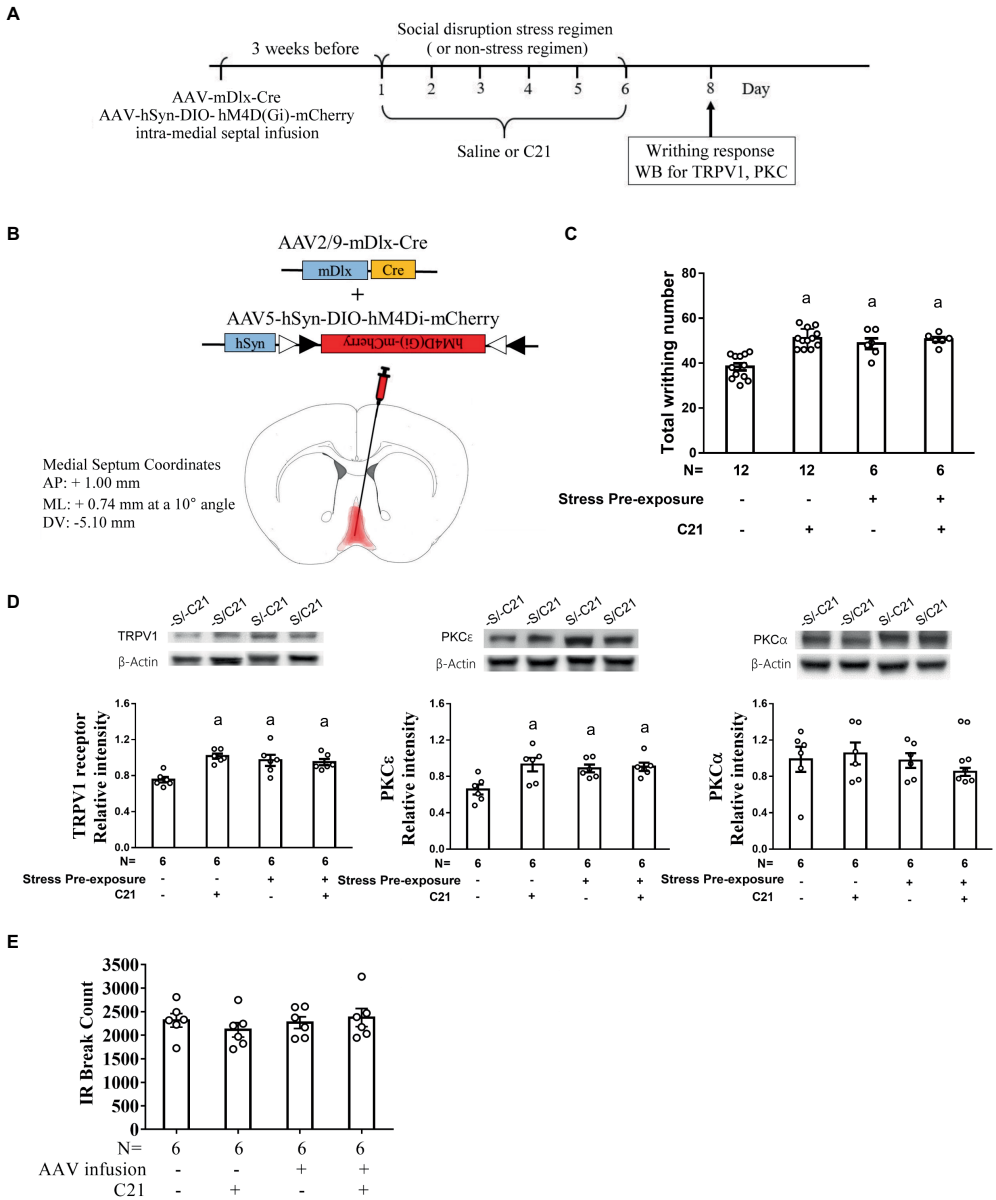
Dampening medial septal GABAergic activity mimicked stress-primed effects on writhing response and accumbal TRPV1 and PKCε level. **(A)** Timeline for viral infection, C21 treatment, the stress regimen, writhing response test and Western assay. **(B)** Intra-medial septal viral mixture infusion. Viral mixture consists of AAV2/9-mDlx-Cre (2.84 × 10^12^ gc/ml) and AAV5-hSyn-DIO-hM4Di-mCherry (2.7 × 10^13^ gc/ml). Superimposed red shaded area indicates all confirmed AAV-mCherry labeling results. **(C)** Total acetic acid-induced writhing responses. ^a^Significantly greater than the –S/–C21 control group. **(D)** Accumbal TRPV1, PKCε and PKCα level. ^a^Significantly greater than the –S/–C21 control group. **(E)** No effects of C21 or intra-medial septal viral infusion on animals’ locomotor activity. Graphs show mean ± SEM.

To rigorously test the determining role of MS GABAergic neuronal activity in mediating stress-primed escalations in writhing magnitude and accumbal TRPV1 and PKCε expression, non-stressed and stressed mice were used. All these mice’ MS GABAergic neurons were exclusively targeted to express membrane hM4Di receptor. One half of stressed and nonstressed mice received C21 treatment (2 mg/kg × 6), while the remaining half from each group received saline treatment (6 injections; Figures 4A,B). A two-way (stress pre-exposure × C21) ANOVA revealed a significant interactive effect of stress pre-exposure and C21 administration on total writhing number [*F*(1,32) = 10.35, *p* = 0.003] (Figure 4C). Six days of social disruption stress was found to produce acid-provoked writhing response escalation, while C21 treatment did not affect such stress-primed writhing escalation [*F*(1,32) = 8.53, *p* = 0.0064] (Figure 4C). Importantly, 6 consecutive days of daily intraperitoneal C21 injection (2 mg/kg each) caused significant escalation of acid-provoked writhes in non-stressed mice [*F*(1,32) = 18.55, *p* = 0.003] (Figure 4C). Moreover, a two-way ANOVA indicated that there was an interactive effect on accumbal TRPV1 expression [*F*(1,20) = 11.58, *p* = 0.0028] (Figure 4D). An escalation in accumbal TRPV1 expression was noticed in non-stressed control mice with C21, but not saline, administration [*F*(1,20) = 8.519, *p* = 0.0085] (Figure 4D). Furthermore, accumbal PKCε level was high in stress-primed mice, while non-stressed mice receiving C21 treatment demonstrated comparable elevation in such accumbal PKCε level [*F*(1,20) = 5.264, *p* = 0.0327] (Figure 4D). Regardless of C21 administration and stress pre-exposure, accumbal PKCα level was unaltered (Figure 4D). It was of importance to note that using these viral mixture methods to inhibit MS GABAergic neural activity did not alter animals’ locomotor activity (Figure 4E). To parcel out the possibility that intra-MS viral mixture infusion and C21 treatment may affect mice’ abdominal pain threshold, an acid dose-dependent curve was obtained. Mice receiving intra-MS viral mixture (hM4Di-mCherry, mDlx-Cre) infusion and C21 treatment had greatest acid-induced writhing magnitude among four groups when acid concentration of 0.9% was used (Figure 3F). However, acid-induced writhes threshold was not altered by such treatment (Figure 3F). To lump together, inactivating MS GABAergic neurons did not affect animals’ general motor activity or their abdominal pain threshold, while produced social disruption stress-like effects, including acid-induced writhing escalation and accumbal TRPV1 and PKCε overexpression, in non-stressed mice.

### 3.4. Dampening dorsal lateral septal cholinergic neuronal activity prevented stress-primed escalations in writhing and accumbal TRPV1 and PKCε expression

To inactivate dorsal LS-accumbal cholinergic projection, an intra-accumbal AAV2/9-ChAT-Cre and intra-dorsal LS AAV5-hSyn-DIO-hM4Di-mCherry intersection strategy was used (Supplementary Figure S3A). Three weeks after viral infusions, 62.1 ± 4.4% of dorsal LS cholinergic neurons were also mCherry-positive, suggesting acceptable infection efficiency by employing this retrograde AAV intersection strategy. Moreover, approximate 82.3 ± 2.8% mCherry-positive cells were cholinergic neurons using such methods, suggesting low off-target effect of intra-dorsal LS and intra-accumbal viral infusion and intraperitoneal C21 treatment (a single dose of 2 mg/kg; Supplementary Figures S3B,C).

**Figure 5:**
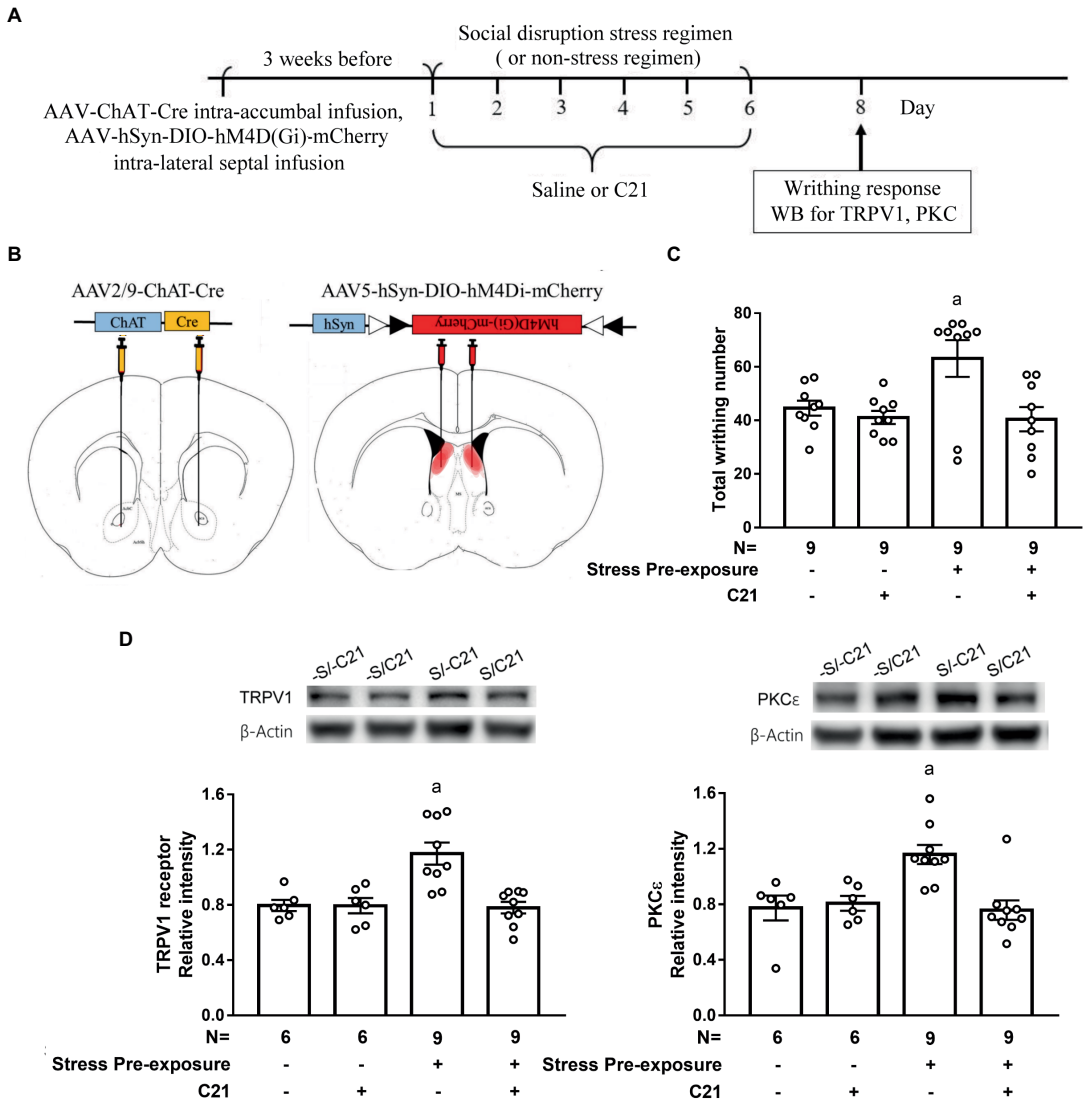
Dampening dorsal lateral septal cholinergic activity prevented stress-primed escalations in writhing response and accumbal TRPV1 and PKCε expression. **(A)** Timeline for viral infection, C21 treatment, the stress regimen, writhing response test and Western assay. **(B)** Intra-dorsal lateral septal (0.3 μl/side, AAV5-hSyn-DIO-hM4Di-mCherry, 2.7 × 10^13^ gc/ml) and accumbal viral (0.3 μl/side, AAV2/9-ChAT-Cre, 2.97 × 10^12^ gc/ml) infusions. Overlayed red shaded area indicates all confirmed AAV-mCherry labeling results. and **(C)** Total acetic acid-induced writhing responses. ^a^Significantly greater than the remaining three groups. **(D)** Accumbal TRPV1 and PKCε level. ^a^Significantly greater than the remaining three groups. Graphs show mean ± SEM.

To assess whether the dorsal LS-accumbal cholinergic projection may be involved in this stress-primed writhing escalation and biochemical plasticity, stressed and non-stressed control mice were used. These mice’ dorsal LS cholinergic neurons were exclusively targeted to express membrane hM4Di receptor (Figures 5A,B). One half of mice in stressed and control mice received C21 (2 mg/kg × 6) treatment, while the remaining halves received equivalent volume of saline treatment. These animals’ acetic acid-provoked writhing responses and accumbal TRPV1 and PKCε assays were done approximately 48 h after the conclusion of the 6-day stress (and C21) regimen (Figure 5A). A two-way (stress pre-exposure x C21) ANOVA revealed a significant interactive effect of stress pre-exposure and C21 administration on acid-induced writhes [*F*(1,32) = 4.532, *p* = 0.0411] (Figure 5C). *Post-hoc* comparisons further indicated that C21 treatment did not alter writhing responses in non-stressed control mice. Six days of stress pre-exposure rendered acid-provoked writhing escalation, while such escalation was prevented by C21 treatment (Figure 5C). Another two-way ANOVA indicated that there was an interactive effect on accumbal TRPV1 expression [*F*(1,26) = 9.723, *p* = 0.0044] (Figure 5D). Importantly, 6-day stress pre-exposure produced significant increases in the TRPV1 expression in nucleus accumbens and such increases were prevented by C21, but not saline, injections (Figure 5D). Finally, accumbal PKCε level was elevated by the stress pre-exposure, while such elevation was also prevented by C21 treatment [*F*(1,26) = 6.311, *p* = 0.0185] (Figure 5D). These results, taken together, suggest that inactivating dorsal lateral septal cholinergic neurons may exert comparable effects as activating medial septal GABAergic neurons on abolishing stress-primed writhing escalation and accumbal TRPV1 and PKCε overexpression.

## 4. Discussion

Using retrograde WGA tract-tracing methods, we noticed that dorsal, but not ventral, lateral septum sent dense projections to both medial and lateral part of the nucleus accumbens. For the first time, we found that medial septal GABAergic neurons sent their projections to the dorsal part of lateral septum. The latter results were in parallel with previous findings (Alonso et al., 1990; Risold and Swanson, 1997; Castañeda et al., 2005) suggesting dorsal, not the ventral, part of lateral septum may be innervated by at least two classes of GABAergic, i.e., somatostatin and parvalbumin mRNA-positive and GAD65- and GAD67-positive, inputs. Moreover, we found that chemogenetic excitation of medial septal GABAergic neurons exerted reliable inhibition on dorsal lateral septal Fos expression. A recent report indicates that curbing local abdominal writhing response did not affect Fos expression in nucleus accumbens (Khoo et al., 2018), suggesting nucleus accumbens may serve as a motor, rather than sensory, anatomical substrate of acid-induced writhes. These results, taken together, prompt us to conclude that medial septal GABAergic inputs are indeed one of the primary input sources to the dorsal lateral septum. Likewise, dorsal lateral septal-accumbal circuit is, at least, a circuit conferring mice’ writhing responses, while reciprocal connections between lateral septum and nucleus accumbens are eminent (Tsanov, 2018).

In our design, intra-medial septum infusion with AAVs in combination was done. And the infection efficiency for AAV2/9-mDlx-Cre and AAV9-hSyn-DIO-hM3Dq-mCherry in combination toward MS GABAergic neuron was as high as 63.2%, suggesting an acceptable infection rate. Moreover, our functional assay revealed dorsal LS Fos expression significantly declined when C21 was given in such AAVs combination-treated mice. However, MS GABAergic efferent may innervate several nuclei other than dorsal LS (Tsanov, 2018). Thus, our results suggest MS GABA neuronal activity at least play a role in modulating social disruption stress-primed escalation in acid-induced writhes and accumbal TRPV1 and PKCε. Furthermore, intra-accumbal AAV2/9-ChAT-Cre and intra-dorsal LS AAV5-hSyn-DIO-hM4Di-mCherry infusions were employed in an attempt to inactivate dorsal LS cholinergic neuronal activity. Using our viral intersection design, AAV5 was expected to proceed intersection with AAV2/9 in dorsal LS, while AAV5 and AAV2 serotypes have been demonstrated to transport along axons in both anterograde and retrograde directions (Haery et al., 2019). Even more so, a proportion of accumbal neurons are indeed ChAT-positive (cholinergic) neurons (Salgado and Kaplitt, 2015). Thus, both dorsal LS and accumbal cholinergic neuronal activity may be involved in social disruption stress-primed plasticity in acid-induced writhing escalation and accumbal TRPV1, PKC over-expression.

Previously, we have demonstrated that medial septal Fos expression seems to correlate with acid-induced writhing magnitude (Liao et al., 2021). In this study, we further found that exciting medial septal GABAergic neuronal activity prevented the stress-primed writhing response escalation, while inhibiting its activity mimicked such stress-primed effects. Moreover, inactivating dorsal lateral septal-accumbal cholinergic projection also prevented the stress-primed writing escalation. These results, taken together, suggest that medial septal GABAergic neuronal hypo-activity and lateral septal cholinergic hyper-activity may serve as biomarkers of choice to predict the stress-primed writhing escalation. Importantly, only medial, but not lateral, septum receives inputs from ventral tegmental area (VTA), while both medial and lateral septum send their projections to VTA (Swanson and Cowan, 1979; Risold and Swanson, 1997). And VTA efferent circuits are regarded as one of the limbic circuits susceptible to both acute and long-lasting stressors (Douma and de Kloet, 2020). For instance, VTA dopaminergic neuronal hyperactivity correlates positively with chronic stress-induced depression-like behavior (Wang et al., 2023). Inhibiting VTA GABAergic neuron may provide long-range disinhibition on many distal brain regions and thus mitigate stress-evoked behaviors (Harlan et al., 2018; Bouarab et al., 2019). Interestingly, inactivating accumbal neuronal activity is found to alleviate morphine withdrawal-induced writhing responses (Wang et al., 2005). Per these lines of evidence, we, hereby, provide a scenario to account for the likely anatomical and neurochemical underpinnings for social disruption stress-primed GABAergic hypo-activity in medial septum. Social disruption stress is suspected to initially render plastic changes in VTA by enhancing VTA GABAergic and/or dopaminergic firing activity. Such heightened GABAergic and dopaminergic activity may exert robust inhibition on medial septal GABAergic efferent. And this medial septal GABAergic efferent hypo-activity is expected to subsequently disinhibit its impact on dorsal lateral septal cholinergic neuron. Consequently, dorsal lateral septal-accumbal cholinergic hyper-activity may excite accumbal efferent and such excited accumbal efferent finally innervates to mesencephalic nuclei being involved in writhing response expression (Groenewegen and Russchen, 1984). Thus, stress-produced neuronal activity plasticity in medial and dorsal lateral septum and nucleus accumbens is sufficient to reflect the stress-primed writhing response escalation. Dampening these stress-produced plastic changes, thus, may effectively abolish stress-primed writhing escalation.

Pharmacological inactivation of the medial septum is report to result in increased or reduced locomotor activity (Osborne, 1994; Oddie, 1998), whereas electrical stimulation or pharmacological activation of MS has little or increased effects on locomotion (Tsanov, 2017; Mocellin and Mikulovic, 2021). These conflicting results are most likely due to traditional cannulation implantation-related tissue deformation and damage (Tsanov, 2018). Sparing cannulation implantation and using a mediolateral angle (10°) of viral injection, chemogenetic excitation of medial septal GABAergic neurons did not seem to produce noticeable apoptosis. Using intraperitoneal C21 administration to excite or inhibit medial septal GABAergic neurons, we demonstrated that non-stressed control mice’ locomotor activity was not altered. That is, those conflicting results of medial septal inactivation and activation on locomotor activity are very likely obtained due to their methods-provoked widespread apoptotic death and/or nonselective drug effect confounds. To conservatively excite or inhibit medial septal GABAergic neuron, we hereby report that such GABAergic activation or inactivation does not seem to affect mice’ locomotor activity, while reliably prevent stress-primed escalation of acid-induced writhing magnitude or produced social disruption stress-like effects on acid-induced writhing escalation.

Performing appropriate and timely behavioral response to reduce further noxious damages, central nervous system is, in theory, informed by the presence of noxious stimuli by its sensory channels at a fast mode. As such, a cationic channel, such as TRPV1 receptor, is not only enriched in many sensory neurons of the pain pathway but fast accessed and activated by various noxious stimuli, including noxious chemicals and heat (Szallasi and Gunthorpe, 2008). Recently, it is reported that protein kinase C (PKC)-mediated TRPV1 phosphorylation in pain sensory pathway seems to be involved in diminished TRPV1 response threshold to inflammatory chemicals (Joseph et al., 2019). A critical implication of the latter finding is that PKC, an intracellular protein kinase, may modulate functional efficacy of TRPV1 receptor and thus enhance the pain magnitude. Noxious chemicals may induce TRPV1 receptor upregulation along with enhanced TRPV1 channel activity (Li et al., 2019). In this study, accumbal TRPV1 and PKCε, rather than PKCα, over-expressions were found exclusively in social disruption stress-experiencing mice. Likewise, these stress-primed mice also had heightened acid-induced writhing response magnitude. A recent study has indicated that intraperitoneal acetic acid injection may induce acute writhes and acute inflammatory cell infiltration in local injection peritoneum (Zhan et al., 2021). In this study, accumbal TRPV1 and PKCε over-expression correlated positively with writing response escalation, suggesting chemical and inflammatory pain may both confer such escalation in writhing magnitude.

Our results demonstrated that exciting medial septal GABAergic neurons did not seem to alter mice’ sensitivity or threshold to intraperitoneal acetic acid stimulation. Nonetheless, we found that exciting medial septal GABAergic neuronal activity prevented stress-primed accumbal TRPV1 and PKCε over-expression and acid-induced writhing escalation. Moreover, inactivating dorsal lateral septal cholinergic neurons prevented stress-primed accumbal TRPV1 and PKCε over-expression and acid-induced writhing escalation. Furthermore, inactivating medial septal GABAergic neuronal activity produced accumbal TRPV1 and PKCε over-expression and acid-induced writhing escalation in naive mice. These results, taken together, suggest that concomitant TRPV1 and PKCε over-expression in nucleus accumbens may be regarded as biomarkers of choice to predict the emotion- and motivation-potentiated abdominal pain magnitude.

## 5. Conclusion

Using retrograde WGA tract-tracing and immunocytochemical staining methods, we have demonstrated the existence of a medial septal-dorsal lateral septal-accumbal projection. Neuronal plasticity in this medial septal-dorsal lateral septal-accumbal projection is sufficient to modulate social disruption stress-primed escalations in acid-induced writhing magnitude and accumbal TRPV1 and PKCε over-expression. Specifically, excitation of medial septal GABAergic neuronal activity may prevent all these stress-primed effects. In contrast, inactivating medial septal GABAergic neurons may produce all these stress-primed effects in non-stressed animals. Finally, dorsal lateral septal and accumbal cholinergic neuronal activity may be involved in these stress-primed escalations.

## Data availability statement

The original contributions presented in the study are included in the article/Supplementary material, further inquiries can be directed to the corresponding authors.

## Ethics statement

The animal study was reviewed and approved by Animal Care Committee at National Cheng Kung University College of Medicine.

## Author contributions

Y-HL, L-HS, W-JY, and LY conceived, designed the experiments, and wrote the paper. Y-HL and Y-CS, conducted the experiments. Y-HL, Y-CS, and L-HS analyzed the data. Y-HL, W-JY, and LY gave final approval of the version to be published. All authors contributed to the article and approved the submitted version.

## Funding

This study is supported by ROC Ministry of Science and Technology (MOST) grants 106-2410-H-006-029-MY3 and 108-2410-H-006-040-MY2 to LY. and 109-2423-H-006-002-MY2 and 111-2423-H-705-001 to Y-HL.

## Conflict of interest

The authors declare that the research was conducted in the absence of any commercial or financial relationships that could be construed as a potential conflict of interest.

## Publisher’s note

All claims expressed in this article are solely those of the authors and do not necessarily represent those of their affiliated organizations, or those of the publisher, the editors and the reviewers. Any product that may be evaluated in this article, or claim that may be made by its manufacturer, is not guaranteed or endorsed by the publisher.
