# Supplementary material for: Medial and dorsal lateral septum involving social disruption stress-primed escalation in acid-induced writhes
Source: Front Mol Neurosci. 2023 Apr 20;16:1158525. doi: 10.3389/fnmol.2023.1158525 (PMC10157398; doi:10.3389/fnmol.2023.1158525)
Supplement: Supplementary file 1 [file Data_Sheet_1.docx]

Supplementary Material

Medial and dorsal lateral septum involving social disruption stress-primed escalation in acid-induced writhes

Yi-Han Liao*, Li-Han Sun, Yi-Chi Su, Wei-Jen Yao*, Lung Yu*

*** Correspondence:** Y-H. Liao: yihanliao@gmail.com; W-J. Yao: 07568@cych.org.tw; L. Yu: lungyu@mail.ncku.edu.tw

## Supplementary Figures


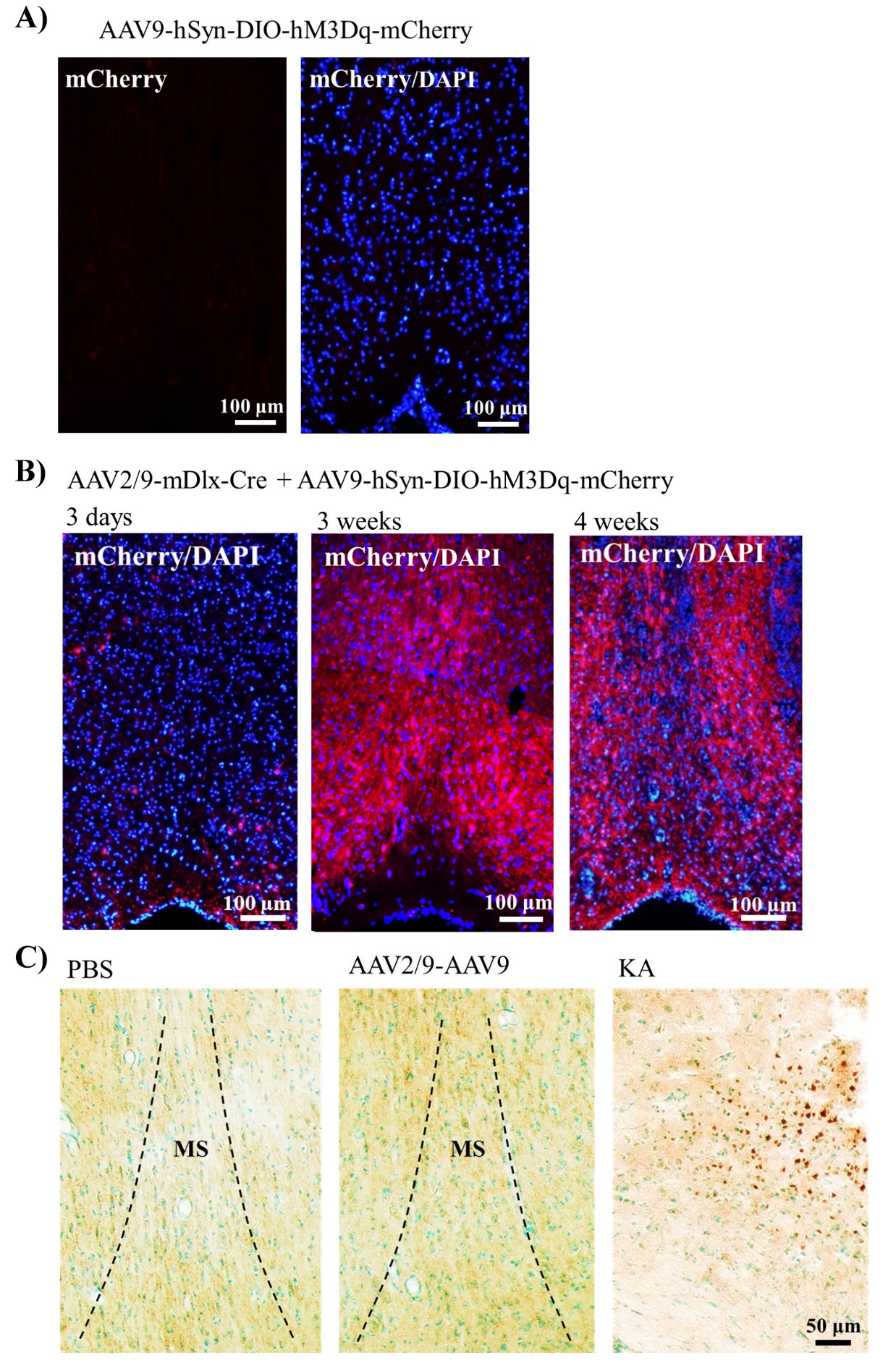


**Figure S1.** **Intra-medial septal infusion protocols were optimized by variant infection times.** A) Representative photomicrographs showing mCherry+ (red) and DAPI+ (blue) spots in intra-medial septal infusion with AAV9-hSyn-DIO-hM3Dq-mCherry (2.4 x 10^13^ gc/ml) alone. Scale bar = 100 μm. B) Representative photomicrographs depicting co-mCherry (red) and DAPI (blue) staining results 3 days, 3 and 4 weeks after the intra-medial septal viral infusions. Scale bar = 100 μm. C) Representative photomicrographs depicting TUNEL assays in intra-medial septal AAV2/9 and AAV9 viral mixture infusion and kainic acid-treated dorsal auditory cortex. MS is the short from of medial septum. Scale bar = 50 μm.


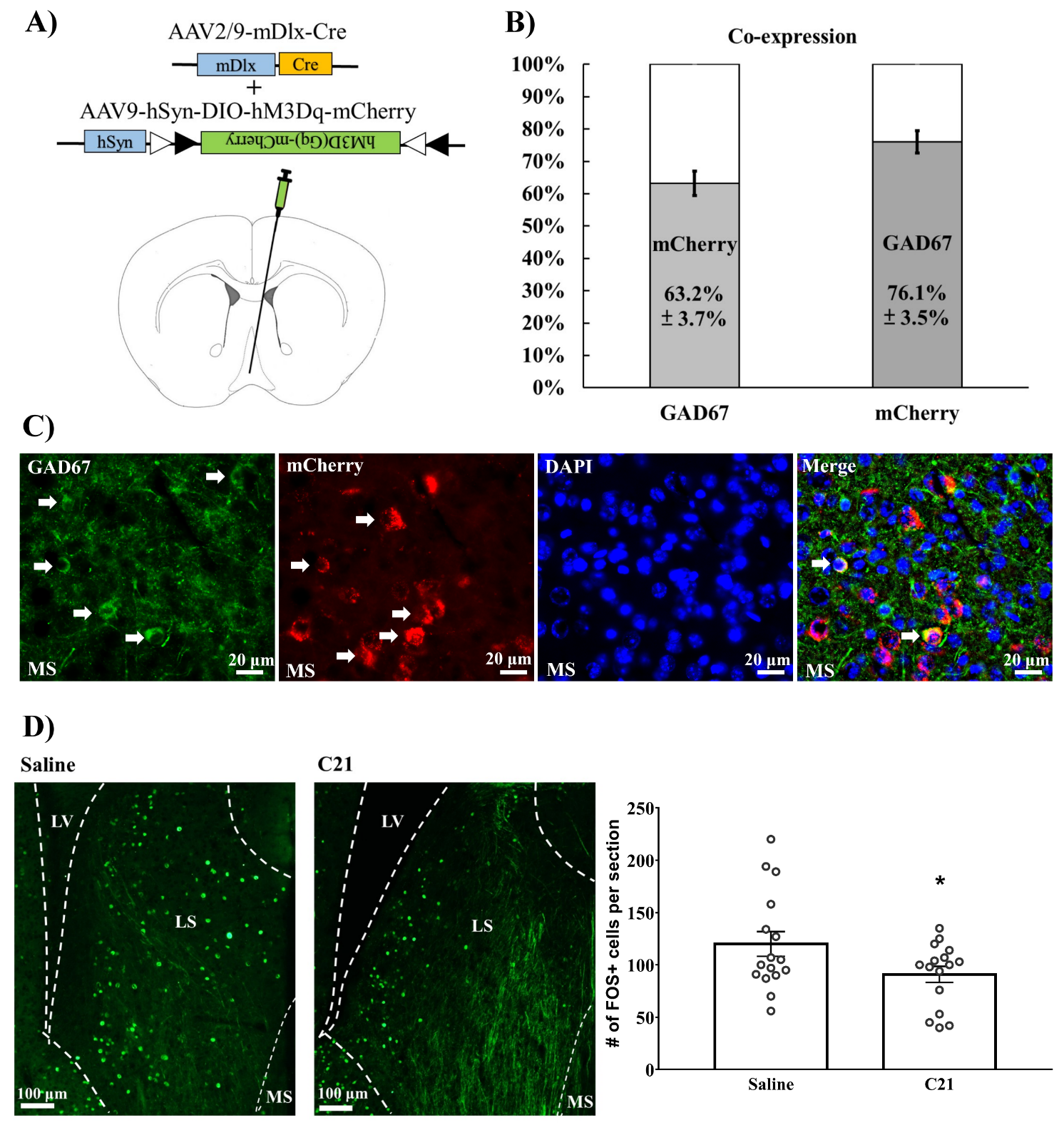


**Figure S2.** **Medial septal viral infection efficiency and FOS-activating effect in dorsal lateral septum were confirmed using intra-medial septal AAV2/9-mDlx-cre and AAV9-hSyn-DIO-hM3Dq-mCherry infusions.** A) Delivery of a mixture of AAV2/9-mDlx-Cre (2.84 x 10^12^ gc/ml) and AAV9-hSyn-DIO-hM3Dq-mCherry (2.4 x 10^13^ gc/ml) into medial septum (anteroposterior: +1 mm, lateral: ±0.74 mm, and dorsoventral: -5.1 mm from bregma) at a 10^o^ angle. B) Venn diagrams for GAD67- and mCherry-expressing neurons. Mean ± SEM is shown. C) GAD67+ (green), mCherry+ (red), DAPI+ (blue), (GAD67/mCherry/DAPI)+ (indigo center, yellow surround) neurons in medial septum. White arrows annotate positive cells. Scale bar = 20 μm. D) Representative photomicrographs showing lateral septal FOS immunostainings in mice receiving intra-medial septal viral mixture infusions and intraperitoneal saline or C21 injection. Graph shows mean ± SEM. *Significantly lower than the other group. LV, LS, and MS are short forms of lateral ventricle, lateral septum, and medial septum. Scale bar = 100 μm.


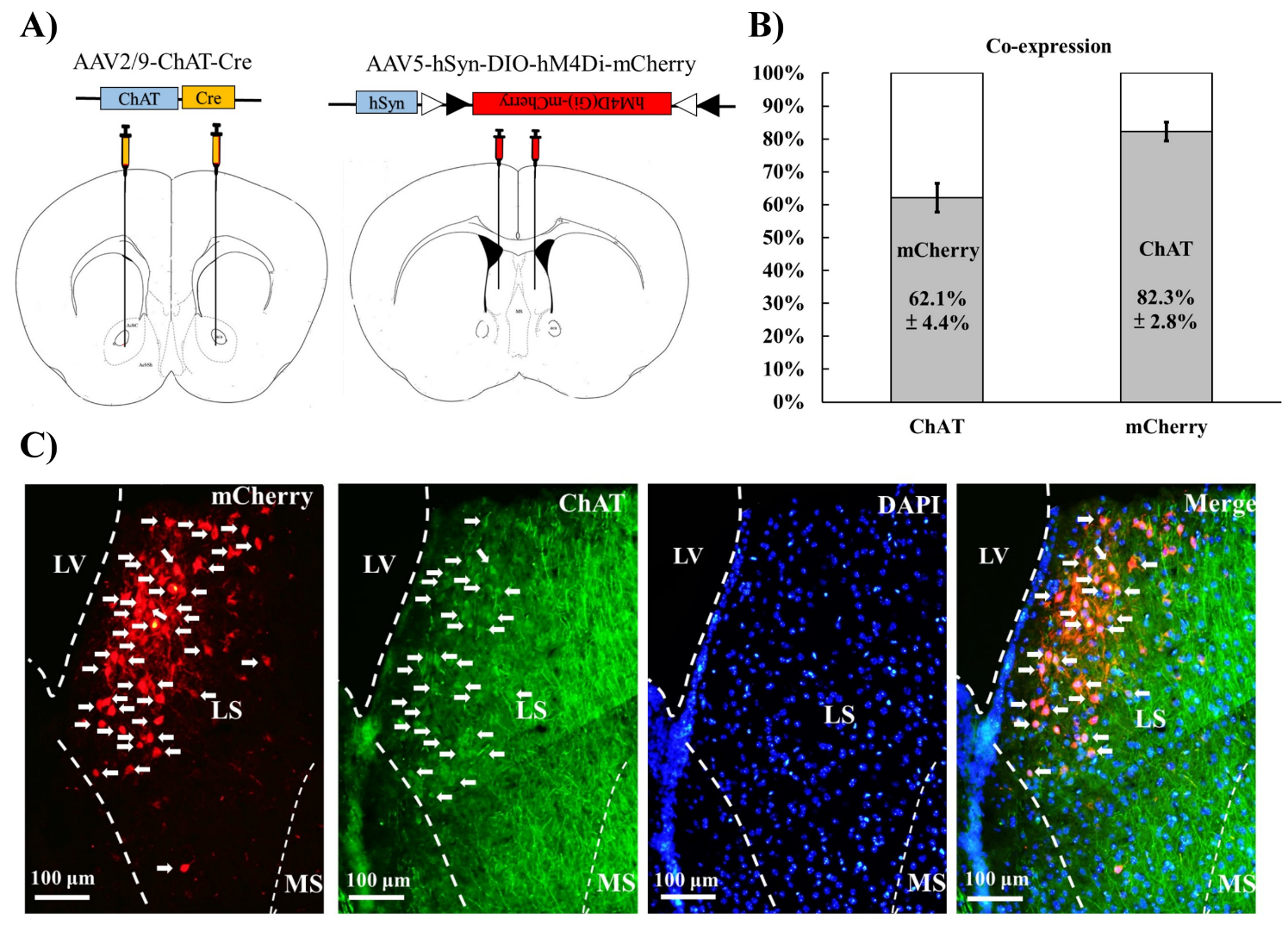


**Figure S3. Dorsal lateral septal viral infection efficiency was confirmed using intra-accumbal AAV2/9-ChAT-Cre and intra-dorsal lateral septal AAV9-hSyn-DIO-hM3Dq-mCherry infusions.** A) Delivery of AAV2/9-ChAT-Cre (2.97 x 10^12^ gc/ml) into accumbal (anteroposterior: +1.34 mm, lateral: ±1.00 mm, and dorsoventral: -4.50 mm from bregma) and AAV5-hSyn-DIO-hM4Di-mCherry (2.7 x 10^13^ gc/ml) into dorsal lateral septum (anteroposterior: +0.6 mm, lateral: ±0.3 mm, and dorsoventral: -3.3 mm from bregma). B) Venn diagrams for ChAT- and mCherry-expressing neurons. Mean ± SEM is shown. C) ChAT+ (green), mCherry+ (red), DAPI+ (blue), (ChAT/mCherry/DAPI)+ (indigo center, yellow surround) neurons in dorsal lateral septum. White arrows annotate positive cells. LV, LS, and MS are short forms of lateral ventricle, lateral septum, and medial septum. Scale bar = 100 μm.
